# Human adipose-derived stem cells partially rescue the stroke syndromes by promoting spatial learning and memory in mouse middle cerebral artery occlusion model

**DOI:** 10.1186/s13287-015-0078-1

**Published:** 2015-05-09

**Authors:** Fei Zhou, Shane Gao, Lin Wang, Chenxi Sun, Lu Chen, Ping Yuan, Haiyang Zhao, Yi Yi, Ying Qin, Zhiqiang Dong, Limei Cao, Haiyan Ren, Liang Zhu, Qiang Li, Bing Lu, Aibin Liang, Guo-Tong Xu, Hongwen Zhu, Zhengliang Gao, Jie Ma, Jun Xu, Xu Chen

**Affiliations:** Neurology Department, Shanghai Eighth People’s Hospital Affiliated to Jiangsu University, Shanghai, 200233 China; East Hospital, Tongji University School of Medicine, Shanghai, 200120 China; Department of Pediatric Neurosurgery, Xinhua Hospital of Shanghai Jiaotong University, Shanghai, 200092 China; Tongji Hospital, Tongji University School of Medicine, Shanghai, 200442 China; Shanghai Xu Hui District Hospital Affiliated to Jiangsu University, Shanghai, 200031 China; Laboratory of Clinical Visual Science, Tongji Eye Institute, Tongji University School of Medicine, Shanghai, 200092 China; Tianjin Hospital, Tianjin, 300211 China; Tianjin Academy of Integrative Medicine, Tianjin, 300100 China; Institute of Translational Medicine, Tongji University School of Medicine, Shanghai, 200092 China; Tenth People’s Hospital Affiliated to Tongji University, Shanghai, 200092 China

## Abstract

**Introduction:**

Growing evidence has brought stem cell therapy to the forefront as new promising approaches towards stroke treatment. Of all candidate seeding cells, adipose-derived stem cells (ADSCs) are considered as one of the most appropriate for stroke treatment. However, previous experimental data could not reach to an agreement on the efficacy of ADSC transplantation for treating stroke *in vivo* as well as its mechanism which hinders their further clinical translational application.

**Methods:**

To explore their *in vivo* mechanism of hADSC administration on neurological injury, hADSC were labeled with Enhanced Green Fluorescence Protein expressing FG12 lentivirus and injected into MCAO mouse infarct area by *in situ* way. Neurological function was evaluated by Rogers Scaling System and their spatial learning and memory was determined by Morris Test. 2,3,5-triphenyltetrazolium chloride was carried out to compare the infarct area among groups. Histoimmunostaining was used to track the injected hADSCs for their *in vivo* migration, transdifferentiation and integration with the endogenous neuronal circuitry. To better address the underlying rescuing mechanism, qRT-PCR was performed on neural markers of MBP, MAP2, GFAP, microglia marker of Iba1.

**Results:**

It was found that hADSCs could promote both spatial learning and memory of MCAO mice. Co-localization of GFP and MAP2 were found in the whole cortex with significantly (P<0.01) higher percentage at the contralateral cortex compared with the ipsilateral cortex. Low percentage of GFP and GFAP co-localized cells were found at whole cortex. Meanwhile, Iba1^+^ microglia and GFAP^+^ astrocyte cells were significantly (P<0.05) suppressed by hADSC injection.

**Conclusions:**

hADSCs could transdifferentiate into neuron like cells (MAP2^+^) *in vivo* and probably used as seeding cells for replacement based stem cell therapy of stroke. Also, significant immunomodulation was found. Meanwhile hADSCs could significantly protect the endogenous neuron survival. This study demonstrated that hADSC intervention with MCAO mice could apparently ameliorate stroke symptoms by direct cell replacement, enhanced immnunosuppression and increasing the viability of endogenous neurons.

**Electronic supplementary material:**

The online version of this article (doi:10.1186/s13287-015-0078-1) contains supplementary material, which is available to authorized users.

## Introduction

Stroke is one of the most devastating diseases and most survivors suffer persistent and severe neurologic deficits including motor, sensory, and cognitive dysfunctions. No treatment for improving the outcome, other than i.v. thrombolysis, has been shown to be effective in clinical practice; however, the narrow therapeutic time window makes thrombolysis treatment a possibility for only a small percentage of patients [1]. Incipient protective therapies have consistently failed as they have been directed towards neural cells, whereas, after a stroke, not only neurons but all components of the neurovascular unit are compromised [2]. These problems represent the major challenges to be overcome by stem cell-replacement therapy.

Several studies in preclinical stroke models have shown that brain damage after stroke not only leads to neuron loss but also to a dramatic decrease of brain plasticity [3] and new therapeutic strategies should focus not only on replenishing lost neurons and promoting endogenous neurogenesis but also on enhancing other related processes, such as gliagenesis, oligodendrogenesis, remyelination, synaptic plasticity, axonal sprouting, and angiogenesis in order to improve neurological function. The presence of stem cells in different structures of the organism suggests that these cells are involved in natural cell-renewal and repair of tissues throughout the lifespan of an individual; therefore, the administration of stem cells seems to modulate these endogenous mechanisms involved in brain plasticity [4]. In this sense, stem cells, in particular mesenchymal stem cells (MSCs), fulfill all these requirements. These cells can be obtained from various tissues and were originally described as adherent cells with a fibroblast morphology and the capability of differentiating into cells of mesodermal origin, such as osteocytes, chondrocytes, and adipocytes [5]. They also show the ability to differentiate into different embryonic layers such as ectoderm and endoderm with excellent self-renewal capacity [6]. Although being initially identified from bone marrow (BMSCs), MSCs have also been successfully isolated from adipose tissue, pancreas, liver, skeletal muscle, dermis, synovial membrane, trabecular bone [7-9], umbilical cord blood [10], lung tissue [11], and dental pulp and periodontal ligament [12]. Adipose-derived stem cells (ADSCs) possess several advantages including abundance, easy accessibility, active self-renewal with low senescence, being free from ethical debate and low immunogenicity, compared with other mesenchymal stem cells and may represent one of the most interesting sources for cell-replacement therapy [13-16]. Comparing the effects of intravenous administration of both BMSCs and ADSCs for stroke therapy in middle cerebral artery occlusion (MCAO) models, both demonstrated significant therapeutic effects but ADSC treatment seems more beneficial [17,18]. Lack of expression of MHC-II in ADSCs also advocates their allogeneic administration and possibly allows ADSCs from healthy and younger donors to be stored in biobanks for the treatment of stroke patients during the acute phase of the disease [4].

The functional outcome of allogeneic ADSC intervention with neurological injury was mainly observed through biomedical studies. Most available reports have demonstrated functional recovery after ADSC injection [17,19-22]. Despite all these beneficial effects of ADSC therapy on stroke outcome, the mechanisms involved in this functional process still remain elusive and should be studied in greater depth.

Previous studies have demonstrated that allogeneic ADSCs can survive in brain parenchyma [23] and express characteristic markers of neurons and glial cells (microtubule-associated protein 2 (MAP2) and glial fibrillary acidic protein (GFAP), respectively) when injected into the lateral ventricle of the rat brain after MCAO [21]. Another study has reported that the introduced exogenous ADSCs expressed endothelial markers (von Willebrand factor (vWF) and endothelial barrier antigen), but not neuronal or glial markers [22]. Nevertheless, the low number of donor cells found in the lesion area could suggest that the ADSCs suffer from apoptotic death or autophagia in the weeks following their administration. It is well-established that cell integration is not enough to explain the improvement in the functional deficits. The beneficial effects of stem cell therapy are probably also mediated by other important mechanisms. In line with these observations, several studies have reported that after intravenous administration, ADSCs were not yet fully implanted into the infarct area [18,20], suggesting that it might not be necessary for stem cells to migrate and graft into the lesion site in order to obtain a positive functional recovery [24].

There is accumulating evidence suggesting that ADSCs could secrete several trophic factors after post-stroke administration, including brain-derived neurotrophic factor (BDNF) [20], insulin-like growth factor-1 (IGF-1) [25], vascular endothelial growth factor (VEGF), and hepatocyte growth factor (HGF) [18], which could be involved in the functional improvement of stroke symptoms in animal models via the reduction in brain injury-derived apoptosis and enhancement of the natural repair response activated by brain injury. Therefore, the secretion of trophic factors by ADSCs could play an important role in reducing cellular apoptosis incurred by cerebral ischemic injury, partly by promoting the expression of Bcl-2, which participates in apoptotic signaling after mitochondrial damage, and by inhibiting the expression of caspase-12, which mediates endoplasmic reticulum stress-induced apoptosis [26]. Moreover, ADSC administration decreased cell death [17,22,23] by preventing nitric oxide release in response to cerebral ischemia [27].

Other authors have analyzed the effects of administration of the cell-free extract from ADSC culture in different injury models, such as oxidative stress and cerebral ischemia. They reported that the cell free complex mixture itself is capable of exerting a protective activity [19,28]. This protective role is observed under different stress conditions including *in vitro* models of cerebral infarct (oxygen and glucose deprivation), and also *in vivo* models (transitory and permanent focal cerebral ischemia and intra-cerebral hemorrhage). More importantly, recent data also suggest that direct communication might occur between the injured brain and administered cells. Furthermore, it has been shown that oxidative stress stimulates the activation of p38 mitogen-activated protein kinases (MAPK) and expression of recombinant human bone morphogenetic protein 2 (BMP2) and basic fibroblast growth factor (bFGF) in human ADSC cultured *in vitro* [29]. This result may indicate that treatment with ADSCs can be considered a ‘living/responding treatment’ that is capable of responding to and modulating the secretion of specific trophic factors under stress environments. *In vitro* study discovered that ADSCs modulate the immune system through suppressing proliferation of stimulated peripheral blood mononuclear cells and inhibiting differentiation of monocyte-derived immature dendritic cells. Moreover, *in vitro* cultures showed a higher level of secretion of the cytokines that have been implicated in the immunomodulatory modes of action of multipotent stromal cells, such as IL-6 and transforming growth factor-1 (TGF-1) [30,31]. In addition, ADSC has been shown to attenuate the levels of IL-18, toll-like receptor (TLR)-4, and plasminogen activator inhibitor-1 (PAI-1) [23]. However, little is known about the immunomodulatory effects of ADSC administration *in vivo* after stroke. These points are surely relevant for recovery and should be analyzed in future research.

In all, previously published data did not reach an agreement on how ADSC works *in vivo*. Different animal models, various ADSC delivery methods (intravenous delivery or *in situ* injection), different ADSC donors (allogeneic or autologous), tracing time (shorter or longer) and ADSC intervention time window (acute or subacute) may largely contribute to the treatment outcome as well as their underlying mechanism. This study chose human ADSCs (hADSCs) as the donor cells to treat a wild type C57/BL6 derived stroke mouse model seven days after surgery by an *in situ* injection method to observe their therapeutic effects at the subacute phase. Immunogenicity, immunomodulation and exogenous cell survival/integration were analyzed. The functional recovery was evaluated by the Rogers Scale system and Morris Maze assay.

## Methods

All animal experiments were performed according to protocols approved by the Ethical Committee of the Experimental Animal Center affiliated with the Medicine School of Tongji University. The approval No. is TJLAC-014-012. hADSCs were isolated from human adipose liposuction liquid, and the processing and handling protocols were approved by the Ethical Committee of the East Hospital Affiliated with Tongji University. The committee director is Fu,Meng. The Approval No. is 2015–045. The human adipose liposuction liquid was obtained from the cosmetic plastic surgery hospital with a consent statement by the patients.

### hADSC isolation, expansion and characterization

hADSCs were isolated from human fresh adipose liposuction liquid according to previously published methods [13,16]. Flow activated cell sorting (FACS, Beckman Coulter) was carried out to identify the CD surface antigen of the isolated hADSCs at passage 3 to 5 according to published protocols [17,32]. Briefly, hADSCs cultured at passage 3 to 5 were first collected by trypsin (0.25%) digestion, washed with flow wash buffer (1 × Dulbecco’s phosphate-buffered saline, 0.5% BSA, and 0.1% sodium azide), and blocked with wash buffer supplemented with mouse immunoglobulin G (25 μg/ml) for 10 minutes on ice. A total of 100 μl of this cell suspension (approximately 5 × 10^5^cells) was aliquoted per tube. Antibodies conjugated with either tricolor fluorescein isothiocyanate (FITC), phycoerythrin (PE), or allophycocyanin (APC) were purchased including CD44-APC (Cat#47-0441-80, eBioscience), CD29-FITC (Cat#11-0299-41, eBioscience), CD105-PE (Cat#12-1057-41, eBioscience), CD73-FITC (Cat#11-0739-42, eBioscience), CD34-FITC (Cat#11-0349-41, eBioscience), CD45-FITC (Cat#11-9459-41, eBioscience). Matched isotype control combinations were performed to identify the specific interaction of antigen and antibody.

### Middle cerebral artery occlusion mouse model

MCAO mouse was achieved through surgery on C57/BL6 mice as previously described [17,24]. Briefly, six- to eight-week old C57/BL6 mice were housed in a controlled SPF level environment (12 hour light/dark cycle at 21C) with free access to water and standard chow diet. Anesthesia was induced by intraperitoneal injection of chloral hydrate (2%, w/v) at 0.15 ml/10 g body weight. A small incision was made above the rhinal fissure to expose and isolate the right common cerebral artery (CCA) and the branch of the cerebral artery. The CCA branch was permanently ligated just before its bifurcation into the frontal and parietal branches with a 9–0 suture. The external common carotid artery was then permanently ligated at two sites and cut off at the middle of the two sites. The internal common carotid artery was temporarily ligated. A breach was made between the bifurcation and the ligation site on the middle cerebral artery to let the embolus (Cat#2634-A4, Beijing Sunbio Biotech Co., Ltd., Beijing, China) infixed into the internal common carotid artery reach to a depth of about 18 mm. This occlusion was kept for 1.5 hours. Re-perfusion was achieved by carefully taking out the embolus to avoid bleeding. Then, the stitching and necessary sterilization were done. Mice receiving surgery were evaluated for their neurological function following the Rogers Scale score system [24,33]. The successful MCAO mice with a score between 2 and 3 were randomly grouped as the PBS control group (MCAO-Ctrl-PBS) and the hADSC injection group (MCAO-hADSC). To confirm the safety of the surgery, a sham group (MCAO-Sham) which underwent blood vessel exposure without occlusion was also included as the normal control.

### hADSC labeling and stereotactic injection into MCAO brain injury area

To trace exogenously introduced hADSCs in the MCAO brain, enhanced GFP (EGFP) expressing lentivirus FG12 was packaged and infected into hADSCs before injection. hADSCs were observed 48 hours after infection under a fluorescent microscope at a wavelength of 488 nm. A total of 80% to 90% percent of hADSCs labeled with green fluorescence was regarded as successful infection. Then, EGFP-hADSCs were collected and adjusted to a final concentration of 1 × 10^9^ cells/ml suspended in PBS. An amount of 5 μl of this cell suspension was injected into the MCAO mouse brain injury area through a glass micropipette and stereotaxic injector (KDS310, Muromachi-Kikai). Briefly, we injected 5 μl cell suspension from three directions within one pole at the infarct lesion. This injection process took six minutes. After injection, the syringe was kept in the brain tissue for one minute to achieve good absorption. In parallel, 5 μl of PBS was injected into the control MCAO group of mice. For the sham group, nothing was done.

### Neurological function evaluation by the Rogers Scaling System

Each mouse was neurologically evaluated with the Rogers Scaling System by two researchers who were blinded to the experimental groups. The scale categories used followed those previously reported [24,33]. The Rogers Scale score functional status was defined as follows: no deficit (0); failure to extend left forepaw (1); decreased grip of the left forelimb when the tail is pulled (2); spontaneous movement in all directions, contralateral circling if pulled (3); circling or walking to the left (4); movement only when stimulated (5); unresponsive to stimulation (6); and dead (7). Scoring was done every week and the statistical analysis was carried out with GraphPad Prism 5.

### Mouse brain infarction area determined with TTC staining

TTC staining was performed as previously described [34]. After the mice were sacrificed, the cerebrum was immediately removed and put in a −4°C refrigerator for 20 minutes. Then it was sliced into six uniform coronal sections after the olfactory bulb was removed. The sections were placed in 2% (W/V in PBS) 2,3,5-triphenyltetrazolium chloride (TTC, Cat# T8877, Sigma) at 37°C in a water bath, then fixed with 4% paraformaldehyde. The normal brain tissue was dyed pink while the infarction area was pale. The infarction area of every six sections was measured by using image analysis software (Scion Image) and the volume of cerebral infarction was estimated by cerebral infarction = the infarction area × thickness/2.

### Mouse spatial learning and memory evaluation by the Morris test

To evaluate the hADSC rescuing function for mouse spatial learning and memory, a Morris water maze equipped with a digital camera was used to determine these important physiological indexes for post-stroke mice. The protocols follow those published by Diederich *et al*. and Vorhees *et al.* [35,36]. To perform a Morris test, a program was set as follows: five day training by putting each mouse in sequence into the water from each of the four directions every day. During this process, the same mouse should be put into the water with an interval of at least 20 mintutes. Also, when the mouse finishes swimming, keeping it warm was a priority. The longest tracing time lasts for 120 seconds. The fifth day repeats the first day program. The sixth day is the test day with the mice starting from the opposite side of the escape platform. The data were recorded and handled with Any-maze software (EthoVision XT7.0, Noldus Information Technology b.v., Netherlands).

### Immunohistochemistry staining to trace hADSC fate

The EGFP-hADSCs were traced by immunohistochemical staining with the neuronal markers MAP2 (Cat#188011, Synaptic Systems), GFAP (Cat#173011, Synaptic Systems) and human nuclear antigen (HNA, Cat#MAB1281) following previously published techniques [13]. Briefly, after the Morris water maze test, mice from each group were sacrificed and underwent intracardial perfusion. Then the brain was removed, fixed with paraformaldehyde and dehydrated with graded sucrose. Brain sections were obtained at 20 μm with a freezing section machine (Leica CM1850) and subjected to histoimmunochemical staining. Whole brain scanning was used to observe the distribution of the EGFP labled hADSCs using a confocal microscope (Leica, SP8). Also, co-localization was carefully observed to determine whether there was any transdifferentiation of hADSCs into neuronal cells.

### qRT-PCR to detect the subtypes of brain cells

To further explore how the introduced hADSCs rescue the mouse brain function, qRT-PCR was used to detect the gene markers Iba1 and GFAP (for microglia cells), NeuN and Synapsin (for neuron cells), and MBP (for oligodendrocytes). Their primer information is listed in Table 1. The protocols were previously published [13].

**Table 1:** **Primer sequence for the related genes**

**Gene symbol**	**Primer sequence**	**Tm (°C)**	**Product size (bp)**
lba1	For 5′-GCCTGCAGACTTCATCCTCT-3′	60	245
	Rev 5′-GACGCTGGTTGTCTTAGGCT-3′		
MBP	For 5′-CTATAAATCGGCTCACAAGG-3′	53	176
	Rev 5′-AGGCGGTTATATTAAGAAGC-3′		
NeuN	For 5′-CCATCCCTTGCCTTTCCCAT-3′	58	170
	Rev 5′-TCTTCTAGGACCCAGCCCTC-3′		
GFAP	For 5′-TGGCCACTGTGAGGCAGAAG-3′	61	181
	Rev 5′-ACCTCCTCCTCGTGGATCTT-3′		
Synapsin	For 5′-GACGGAAGGGATCACATCAT-3′	57	63
	Rev 5′-CTGGTGGTCACCAATGAGC-3′		
GAPDH	For 5′-CAAGATCATCAGCAATGCCTCCTG-3′	66	363
	Rev 5′-GCCTGCTCACCACCTTCTTGA-3′		

### Statistical analyses

All data are expressed as mean ± standard deviation (SD) or standard error of the mean (SEM). The statistical significance was determined using one-way or two-way analysis of variance (ANOVA) Bonferroni post-tests between groups by GraphPad Prism version 5.00 for Windows (GraphPad Software, San Diego CA, USA). *P*<0.05 was considered statistically significant.

## Results

### hADSC morphology and CD surface antigen profiling

**Figure 1:**
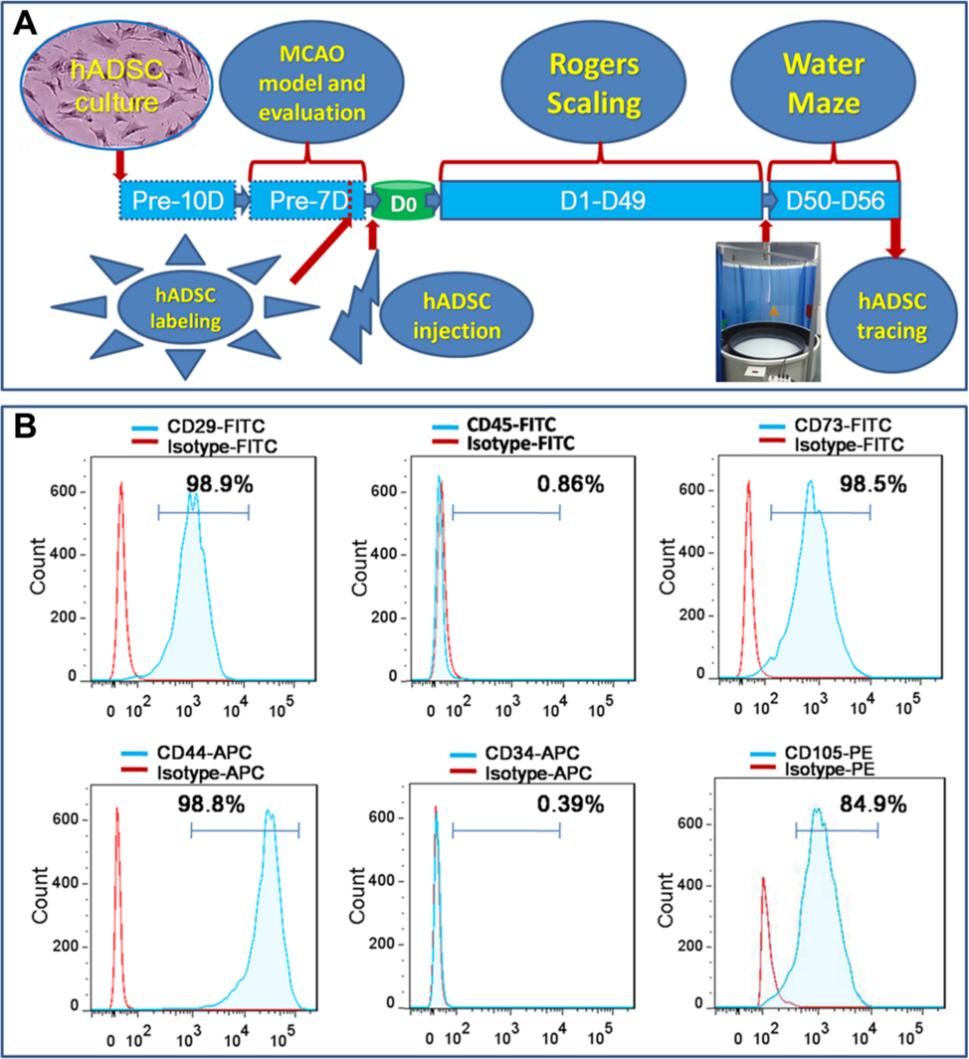
Experimental diagram **(A)**, hADSC surface antigen profiling with FACS showed that hADSCs positively express CD29, CD44, CD73 and CD105, negatively express CD34 and CD45 **(B)**. FACS, flow activated cell sorting; hADSCs, human adipose-derived stem cells.

Isolated hADSCs possess typical spindle-like morphology after passage 3 to 5 as shown in Figure 1A. The experimental design diagram is shown in Figure 1A. The CD surface antigen expression pattern analyzed by flow Jo software after FACS is shown in Figure 1B. More than 84.9% of the hADSCs express MSC CD markers, CD29, CD44, CD73 and CD105, while no more than 0.86% of the hADSCs express the hematopoietic stem cell markers, CD34 and CD45.

### Generation and characterization of MCAO mouse model (stroke model)

**Figure 2:**
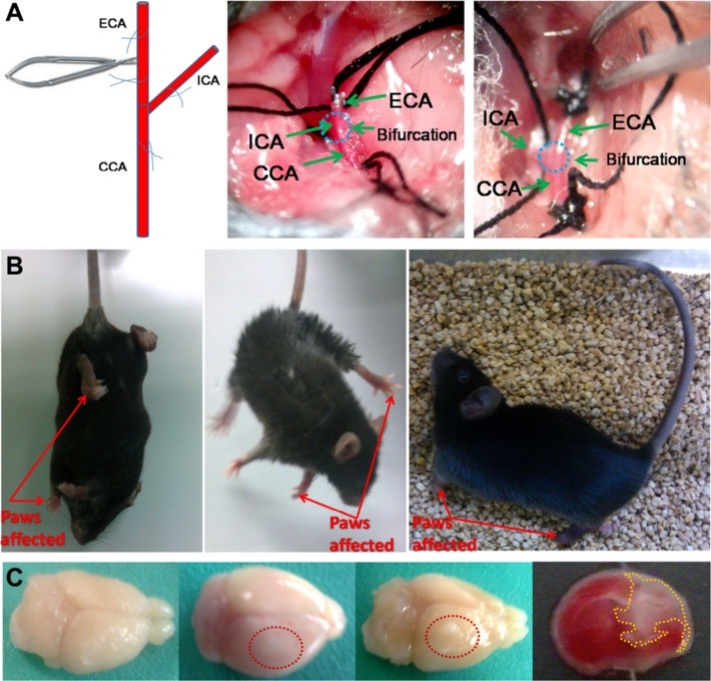
MCAO mouse model processing diagram **(A)**, MCAO mouse model confirmation by abnormal behaviors **(B)**, brain injury appearance and TTC staining was used to confirm the success of the MCAO mouse model **(C)**. MCAO, middle cerebral artery occlusion.

The MCAO mouse model was made following a surgical operation as shown in Figure 2A. The mice manifested weakness in the contralateral limbs after the MCAO model was established. The representative images demonstrating the abnormal behaviors are shown in Figure 2B. To determine the area of the brain damaged by infarction seven days after MCAO, TTC staining was performed and the circled pale color as shown in Figure 2C indicates the infarcted area. TTC staining revealed significant damage to the brain, consistent with the abnormal behavior. These data collectively confirmed the success of the MCAO mouse model. According to the Rogers Scale scoring, those mice receiving MCAO surgery scaled as 2 to 3 scores were randomly assigned to two groups: one used as the control group with PBS injection only named MCAO-Ctrl-PBS (n = 7), the other used as the cell injection group named MCAO-hADSC (n = 7).

### Functional improvements achieved by hADSC transplantation in MCAO mice

**Figure 3:**
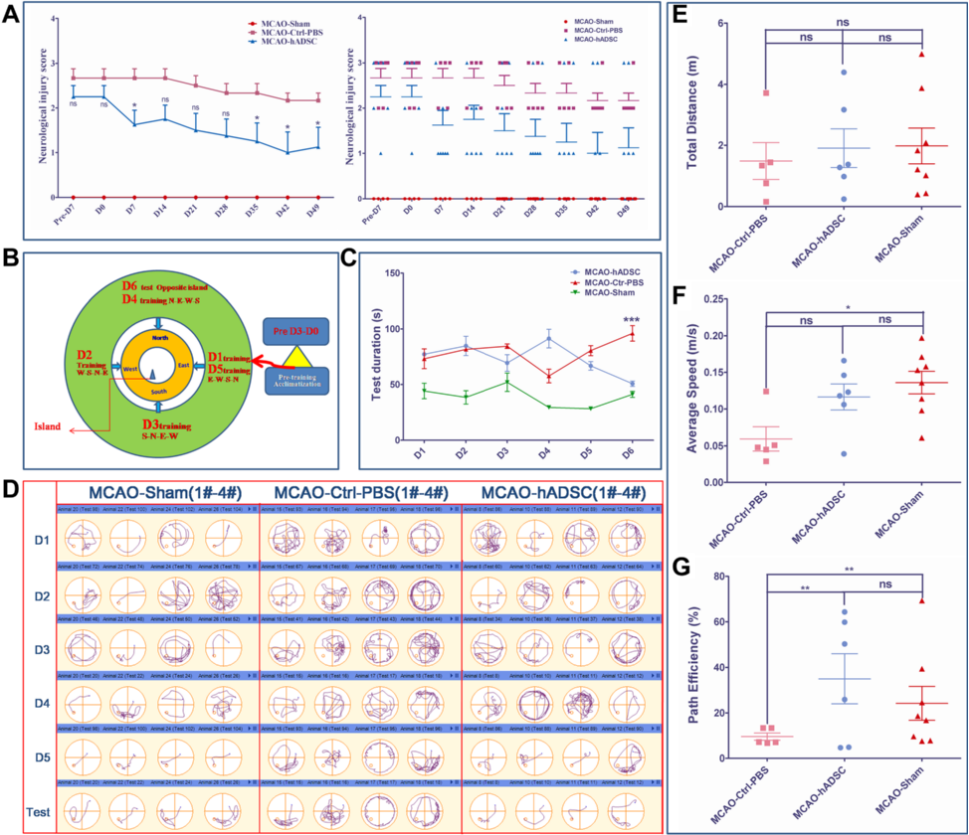
Rogers Scale scoring for three groups of mice and the score distribution within each group **(A)**, water maze performing protocol diagram and the test duration (S) for each group of mice **(B** and **C)**, the water maze track path information for the representative mice within each group **(D)**, their total distance travelled (m), their average speed (m/s) and their path efficiency (%) on the test day (D6) **(E** to **G)**. *,** and *** indicate that significant differences were found between the groups (*P* <0.05, *P* <0.01 and *P* <0.001, respectively).

Seven days after the MCAO surgery, EGFP-hADSCs or PBS solution was stereostatically injected into the infarction area of the MCAO mouse brain. Their behaviors were observed carefully and the score was recorded every week according to the protocol diagram shown in Figure 1A. The Rogers Scale scoring results are shown in Figure 3A. Significant differences (*P*<0.05) could be found between the MCAO-Ctrl-PBS group and the MCAO-hADSC group at 7 days, 35 days, 42 days and 49 days after treatment. To better demonstrate the individual differences within each group, the dotting distribution is also shown in Figure 3A for reference. Seven weeks after hADSCs injection, each group of mice was subjected to the Morris Test. The experimental diagram of the Morris Water Maze is shown in Figure 3B, the water maze test duration (S) is shown in Figure 3C and the tracking path information of representative mice in each group is shown in Figure 3D (the tracking paths of four representative mice for each group are shown). After five days of training, the mice were subjected to the final test. The MCAO-hADSC group mice regained the ability to memorize the target and took a nearly equal time (*P*>0.05) to find the escape platform in as efficient a way as the MCAO-Sham group mice (*P*>0.05) (Figure 3C, D, G), indicating a significant rescuing effect. The MCAO-hADSC group mice took a significantly (*P*<0.001) shorter time to search around and find the target compared with the MCAO-Ctrl-PBS group mice (Figure 3C). Comparing the path length among each group, mice in the MCAO-hADSC group went through a shorter path length and took less time to reach the rescuing platform than those in the MCAO-Ctrl-PBS group at a similar swimming speed (Figure 3E-G). These results indicate that movement ability did not hinder their latent time. Carefully observing the latent process, mice in the MCAO-hADSC group were always more efficient in reaching the rescue platform than those in MCAO-Ctrl-PBS group, but were similar to those in the MCAO-Sham group; the data are shown in Figure 3D and Additional file 1: Video 1, Additional file 2: Video 2, Additional file 3: Video 3, Additional file 4: Video 4, Additional file 5: Video 5, Additional file 6: Video 6, Additional file 7: Video 7, Additional file 8: Video 8 and Additional file 9: Video 9. These data demonstrate that hADSCs transplantation apparently improved the spatial learning and memory of the MCAO mouse indicating their promising therapeutic effects on post-stroke patients.

### Infarction volume was reduced by hADSC administration

**Figure 4:**
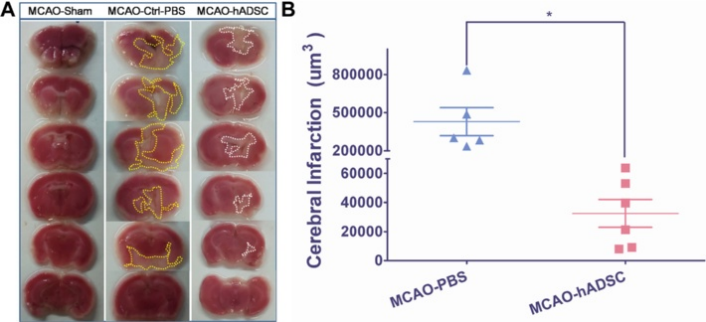
Brain infarction volume determined by TTC staining. Representative TTC staining pictures of mouse brain within each group **(A)**, and the statistical analysis by Scion Image Software **(B)**. * means significant differences were found between groups (*P* <0.05). TTC, 2,3,5-triphenyltetrazolium chloride.

TTC staining was used to determine the brain infarction area. The representative pictures are shown in Figure 4A. The infarction volume obtained statistically with Scion Image is shown in Figure 4B. The infarction volume was significantly (*P*<0.05) reduced in the MCAO-hADSC group compared with the MCAO-Ctrl-PBS group, which indicates the apparent treatment effects, at least in the physical and appearance levels. This result was consistent with those previously reported [17,23,24].

### Transplanted hADSCs transdifferentiated into neuron-like cells *in vivo*

**Figure 5:**
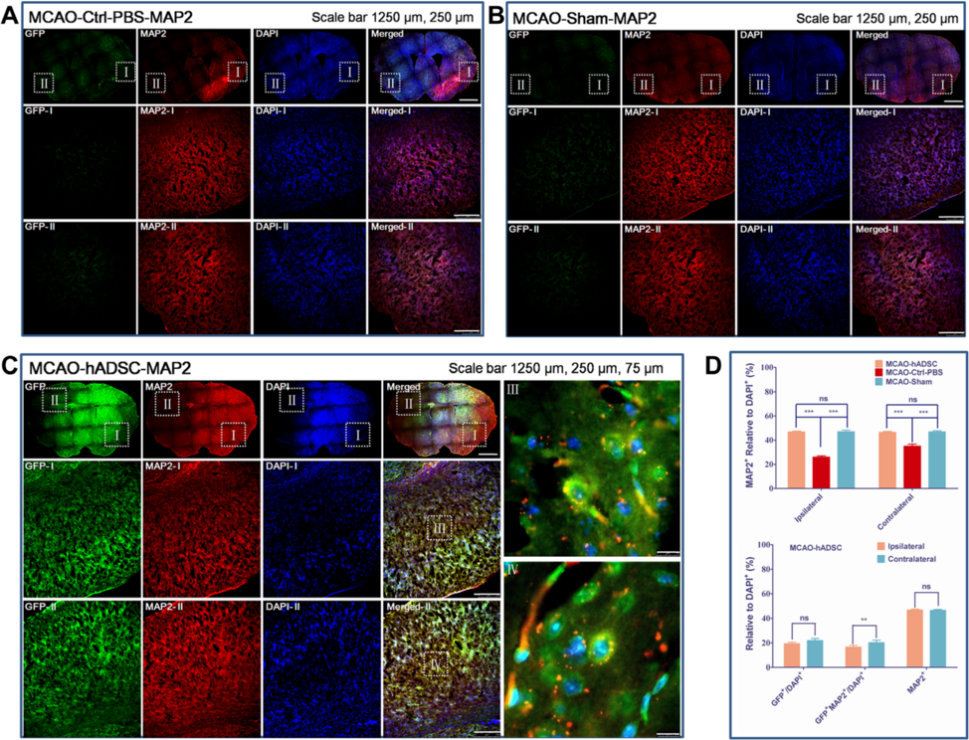
hADSC tracing by histoimmunostaining with MAP2 antibody. MCAO mouse group with PBS injection instead of hADSC serving as the negative control (MCAO-Ctrl-PBS) **(A)**, normal mice with blood vessel exposure only but not occlusion (MCAO-Sham) **(B)**, MCAO mouse group with hADSC injection seven days after MCAO surgery (MCAO-hADSC) **(C)**. Quantitative analysis of GFP labeled hADSCs, MAP2+ relative to the DAPI+ means neurons relative to the total cells in both the ipsilateral and contralateral areas of different groups of the MCAO brain, GFP relative to the DAPI+ means the survival hADSCs take the ratio of total brain cells, GFP+/MAP2+ relative to DAPI+ means the injected hADSCs which differentiated into MAP2+ cells take the ratio of total brain cells **(D)**. *, ** and *** indicates that significant differences were found between groups (*P*<0.05, *P*<0.01 and *P*<0.001, respectively ). DAPI, 4',6-diamidino-2-phenylindole; hADSC, human adipose-derived stem cells; MAP2, microtubule-associated protein 2; MCAO, middle cerebral artery occlusion.

**Figure 6:**
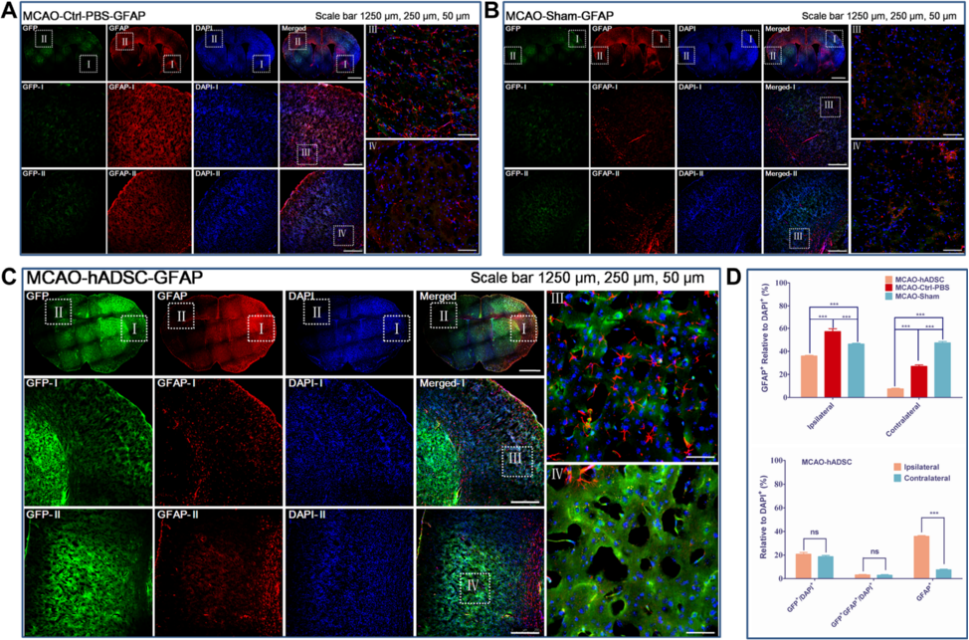
hADSC tracing by histoimmunostaining with GFAP antibody. MCAO mouse group with PBS injection instead of hADSC serving as the negative control (MCAO-Ctrl-PBS) **(A)**, normal mice with blood vessel exposure only but not occlusion (MCAO-Sham) **(B)**, MCAO mouse group with hADSC injection seven days after MCAO surgery (MCAO-hADSC) **(C)**. Quantitative analysis of GFP labeled hADSCs, GFAP+ relative to the DAPI+ means astrocytes cells take the ratio of total cells in both the Ipsilateral and Contralateral of different groups of the MCAO brain, GFP relative to the DAPI+ means the survival hADSCs take the ratio of total brain cells, GFP + /GFAP + relative to DAPI + means the hADSCs transdifferentiated into astrocytes take the ratio of total brain cells **(D)**. *, ** and *** indicates that significant differences were found between groups (*P*<0.05, *P*<0.01 and *P*<0.001, respectively). DAPI, 4',6-diamidino-2-phenylindole; GFAP, glial fibrillary acidic protein; hADSC, human adipose-derived stem cells; MCAO, middle cerebral artery occlusion.

Histoimmnochemical staining was used to trace the migration, transdifferentiation and integration of the introduced hADSCs *in vivo*. The staining pictures and the statistical analysis with neuronal markers MAP2 and GFAP are shown in Figures 5 and 6 and Additional file 10: Figure S1 and Additional file 11: Figure S2 respectively. Neuron-like cells could be found as shown in Additional file 10: Figure S1. Statistical analysis found that a similar (*P*>0.05) percentage of GFP positive cells existed in both the contralateral brain area and the ipsilateral brain area, which indicated their robust *in vivo* migration and survival ability (Figure 5C and D). Moreover, co-localization of GFP and MAP2 was found at a relatively higher (*P*<0.01) percentage in the contralateral brain area indicating the neuron differentiation of introduced hADSCs in a preferred *in vivo* environment because the injured ipsilateral area might not favor this *in vivo* transdifferentiation (Figure 5D). Meanwhile, a significantly higher (*P*<0.001) percentage of MAP2 positive cells was found between the MCAO-hADSC and MCAO-Ctrl-PBS, also between the MCAO-Sham and MCAO-Ctrl-PBS in both of the two brain spheres, but this significant difference was not found between MCAO-hADSC and MCAO-Sham in both of the two brain spheres. On the other hand, co-localization of GFP and GFAP was rarely found, implying a minor transdifferentiation preference of hADSCs to GFAP^+^ cells (Figure 6C and D), which agreed with previously published results [13]. At the same time, it was observed that the introduced hADSCs could significantly (*P*<0.001) suppress the reaction of GFAP expressing cells in both brain spheres compared with their counterparts in the PBS injection control group, implying their anti-inflammation effects (Figure 6D). Colocalization of HNA and GFP could be observed indicating that the GFP positive cells were indeed the introduced hADSCs (Additional file 11: Figure S2).

### hADSCs protect the endogenous neurons and oligodendrocytes while suppressing microglia cells

**Figure 7:**
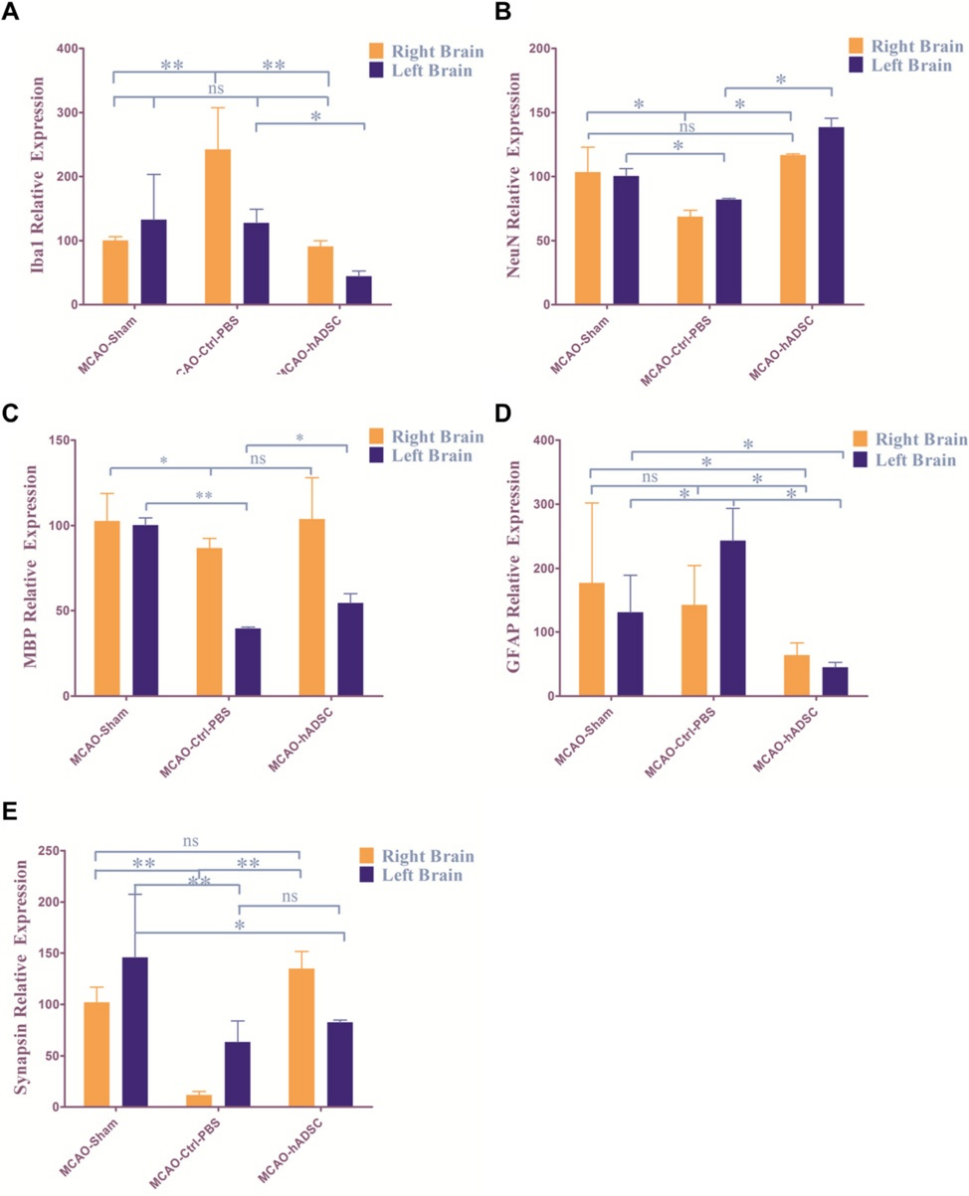
qRT-PCR to detect different endogenous cell marker expression: **(A)** Iba1 for microglia cells, **(B)** NeuN for neurons, **(C)** MBP for oligodendrocytes, **(D)** GFAP for astrocytes, **(E)** Synapsin for neurons. *, ** and *** indicate that significant differences were found between groups (*P*<0.05, *P*<0.01 and *P*<0.001, respectively). GFAP, glial fibrillary acidic protein.

qRT-PCR data indicated that hADSC administration can significantly suppress the expression of the microglia marker Iba1 (*P*<0.01) and the astrocyte marker GFAP (*P*<0.05) (Figure 7A and D) compared with the PBS control group. This demonstrated that hADSCs can decrease brain inflammation by suppressing the reaction of microglia cells caused by damage. At the same time, hADSCs can significantly (*P*<0.05) improve the neuron related genes NeuN and Synapsin (Figure 7B and E) compared to the PBS injected control group mice. This implies that introduced hADSCs can protect the endogenous neuron from damage. Also, the oligodendrocyte marker MBP was significantly (*P*<0.05) improved compared with the PBS control group. These accumulated data support the hypothesis that hADSCs administration can apparently improve the endo-environment and protect the neurons and oligodendrocytes from damage-derived cell death.

## Discussion

hADSCs have been regarded as one of the most promising stem cell candidates to be translated into clinical therapies for many hard-to-treat diseases. Stroke patients have faced medical and economic burdens due to limited available treatments. Stem cell based replacement therapy became attractive because of its potential to thoroughly resolve these problems. hADSCs possess multipotency properties and can be differentiated into neuron cells as demonstrated by many authors [7,13,21]. Characterization of isolated cells indicated that these isolated and expanded spindle like cells at passage 3 to 5 were mostly (>84.9%) composed of adipose derived mesenchymal stem cells. This type of CD antigen profiling is similar to the requirement for MSCs and adipose-derived mesenchymal stem cells [37]. Based on our previous findings with regard to hADSC treatment of traumatic brain injury (TBI) in mice, hADSCs could survive in mouse brain and reduce the injury lesion [13]. This study demonstrated that the infarction area was reduced in MCAO mouse brain and mouse memory and spatial learning were significantly improved, which is consistent with the previously published results [17,23,24]. Our results indicated that hADSC injection may promote endogenous neuron survival after stroke damage, which is also consistent with previously published results [21,25-27,38]. hADSC transplantation can significantly suppress the reaction of GFAP and Iba1 expressing cells indicating their immunomodulation. These results are consistent with previously published findings [22,23,39]. Current data on the efficacy of animal treatment using hADSCs differ with each other due to different animal models, various cell delivery methods and the time window of cell introduction [17,24]. In our study, we injected hADSCs into the mouse brain lesion using an *in situ* method seven days after MCAO surgery. We could trace the injected GFP labled hADSCs and observed GFP and MAP2 double positive cells. This agrees with previously published data [13,21,23]. With qRT-PCR, we further demonstrated that hADSC injection could promote the survival of endogenous neuron and oligodendrocyte cells and modulate the brain immune response. These results agree with [14,17]. Although scientists have made diligent efforts to determine how ADSCs rescue the brain function of ischemic mice, many doubts still exist including whether hADSC derived neurons can integrate into the neuronal circuitry, how the hADSCs modulate the immune response to brain damage, and so on. Further exploration should be performed to make full use of ADSCs as the seeding cells for various neurological diseases.

### Conclusions

Presently, MSCs therapy is becoming a promising new therapeutic option for stroke. Of all MSCs, ADSCs are of special interest. Our results demonstrate that hADSC treatment significantly promotes the spatial learning and memory of MCAO mice. Meanwhile, the infarction volume of MCAO mice could also apparently be reduced implying promising clinical treatment effects. Interestingly, *in situ* introduced hADSCs were partially transdifferentiated into MAP2 positive neuron-like cells with minor GFAP positive ones in the *in vivo* microenvironment. This result implies that probable stem cell replacement based therapy has occurred. Meanwhile, injection of hADSCs could significantly promote the survival of damaged neurons and oligodendrocyte cells. Moreover, significantly decreased expression of GFAP and Iba1 in the MCAO-hADSC mouse group compared with the MCAO-Ctrl-PBS mouse group showed that an immunomodulatory function should occur due to hADSC injection at the subacute phase of stroke disease. Ongoing clinical trials should provide more useful and attractive new information on the safety and efficacy of hADSC for stroke patients in the very near future.

## Additional files

Additional file 1: Video 1. MCAO-Sham water maze track on D1 (D1-Sham-20). Additional file 2: Video 2. MCAO-Sham water maze track on D5 (D5-Sham-20). Additional file 3: Video 3. MCAO-Sham water maze track on D6-test(Test-Sham-20). Additional file 4: Video 4. MCAO-Ctrl-PBS water maze track on D1 (D1-MCAO-PBS-16). Additional file 5: Video 5. MCAO-Ctrl-PBS water maze track on D5 (D5-MCAO-PBS-16). Additional file 6: Video 6. MCAO-Ctrl-PBS water maze track on D6-test (Test-MCAO-PBS-16). Additional file 7: Video 7. MCAO-hADSC water maze track on D1 (D1-MCAO-hADSC-10). Additional file 8: Video 8. MCAO-hADSC water maze track on D5 (D5-MCAO-hADSC-10). Additional file 9: Video 9. MCAO-hADSC water maze track on D6-test (Test-MCAO-hADSC-10). Additional file 10: Figure S1. GFP positive cells showed neuron like morphology. Additional file 11: Figure S2. Histoimmunostaining with human nuclear antigen (HNA) to trace the transplanted hADSCs in mouse MCAO model brain.

## Abbreviations

APC: allophycocyanin
BDNF: brain-derived neurotrophic factor
BMSCs: bone marrow stem cells
bp: base pair
BSA: bovine serum albumin
EGFP: enhanced green fluorescence protein
FACS: flow activated cell sorting
FITC: fluorescein isothiocyanate
GFAP: glial fibrillary acidic protein
hADSCs: human adipose-derived stem cells
HGF: hepatocyte growth factor
HNA: human nuclear antigen
IGF-1: insulin-like growth factor-1
IL: interleukin
MAP2: microtubule-associated protein 2
MCAO: middle cerebral artery occlusion
MSCs: mesenchymal stem cells
PBS: phosphate-buffered saline
PE: phycoerythrin
TTC: 2,3,5-triphenyltetrazolium chloride
VEGF: vascular endothelial growth factor
