# Correction to: Human adipose-derived stem cells partially rescue the stroke syndromes by promoting spatial learning and memory in mouse middle cerebral artery occlusion model

**DOI:** 10.1186/s13287-019-1198-9

**Published:** 2019-03-06

**Authors:** Fei Zhou, Shane Gao, Lin Wang, Chenxi Sun, Lu Chen, Ping Yuan, Haiyang Zhao, Yi Yi, Ying Qin, Zhiqiang Dong, Limei Cao, Haiyan Ren, Liang Zhu, Qiang Li, Bing Lu, Aibin Liang, Guo-Tong Xu, Hongwen Zhu, Zhengliang Gao, Jie Ma, Jun Xu, Xu Chen

**Affiliations:** 1 Neurology Department, Shanghai Eighth People’s Hospital Affiliated to Jiangsu University, Shanghai, 200233 China; 2 East Hospital, Tongji University School of Medicine, Shanghai, 200120 China; 3 Department of Pediatric Neurosurgery, Xinhua Hospital of Shanghai Jiaotong University, Shanghai, 200092 China; 4 Tongji Hospital, Tongji University School of Medicine, Shanghai, 200442 China; 5 Shanghai Xu Hui District Hospital Affiliated to Jiangsu University, Shanghai, 200031 China; 6 Laboratory of Clinical Visual Science, Tongji Eye Institute, Tongji University School of Medicine, Shanghai, 200092 China; 7 Tianjin Hospital, Tianjin, 300211 China; 8 Tianjin Academy of Integrative Medicine, Tianjin, 300100 China; 9 Institute of Translational Medicine, Tongji University School of Medicine, Shanghai, 200092 China; 10 Tenth People’s Hospital Affiliated to Tongji University, Shanghai, 200092 China

**Correction to: Stem Cell Res Ther**

**https://doi.org/10.1186/s13287-015-0078-1**

The original article [1] contains an accidental omission in the Acknowledgements. The corrected Acknowledgements section is shown ahead:

**Acknowledgements**

This work was supported by the Ministry of Science and Technology of China (2010CB945200, 2011CB966200); the National Natural Science Foundation of China (31471029, 81472141, 81070910, 81200832, 81271382); National program for support of Top-notch young professionals (J.X.); Shanghai City, the medical cooperation projects (11DZ192130C); Projects of International Cooperation and Exchanges NSFC (81261130318); the Fundamental Research Funds for the Central Universities; Tongji University Talents Training Program (2011KJ051); New Century Excellent Talents in University (NCET-10-0606); 51th China Postdoctoral surface Project (2012 M510890); Shanghai Postdoctoral Research Funding Scheme of 2012 (12R21416200).
