# Supplementary figures and images for: Human adipose-derived stem cells partially rescue the stroke syndromes by promoting spatial learning and memory in mouse middle cerebral artery occlusion model
Source: Stem Cell Res Ther. 2015 May 9;6(1):92. doi: 10.1186/s13287-015-0078-1 (PMC4453264; doi:10.1186/s13287-015-0078-1)

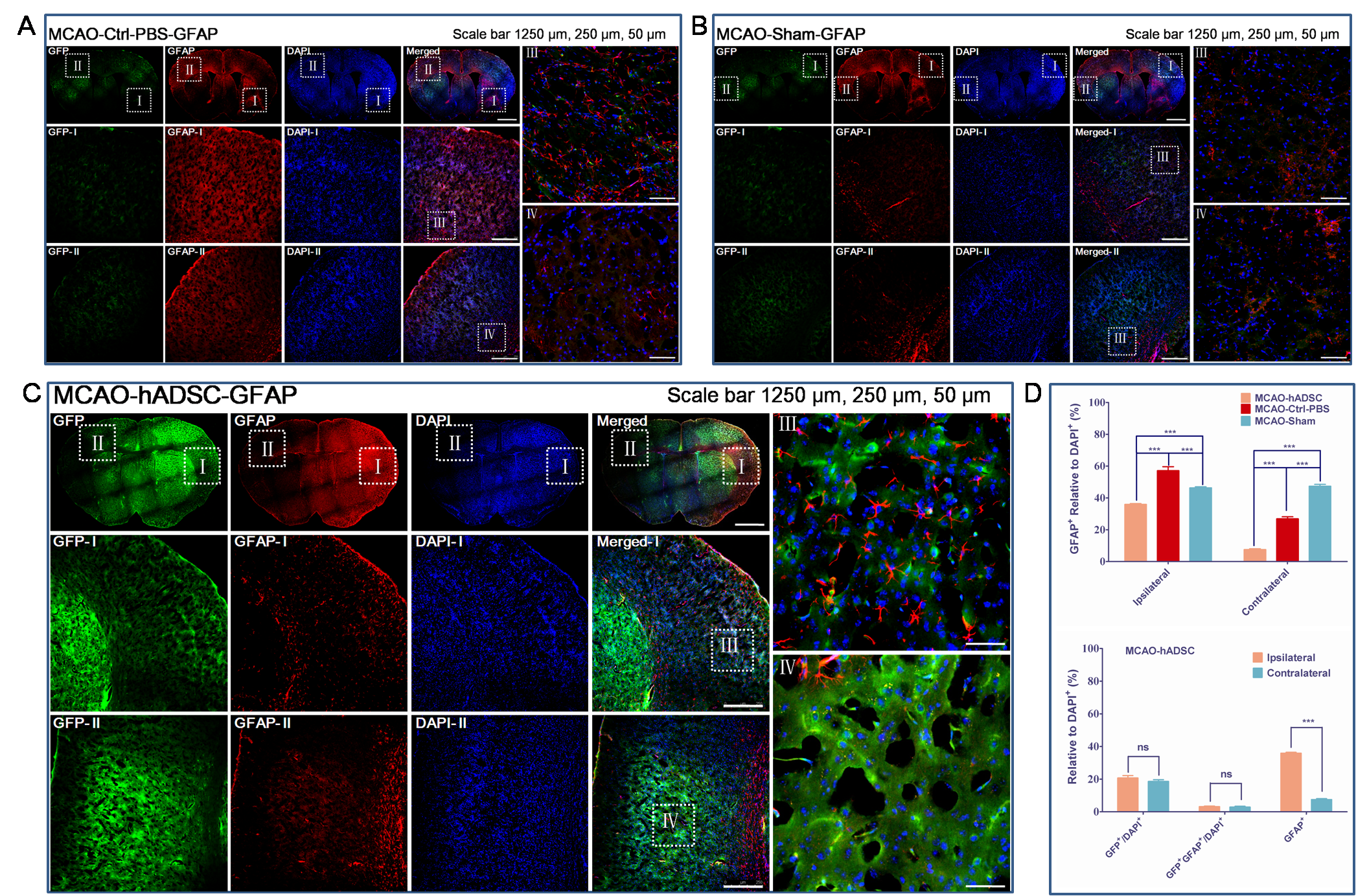

Supplement: Additional file 10: Figure S1. — GFP positive cells showed neuron like morphology. [file 13287_2015_78_MOESM10_ESM.tiff]

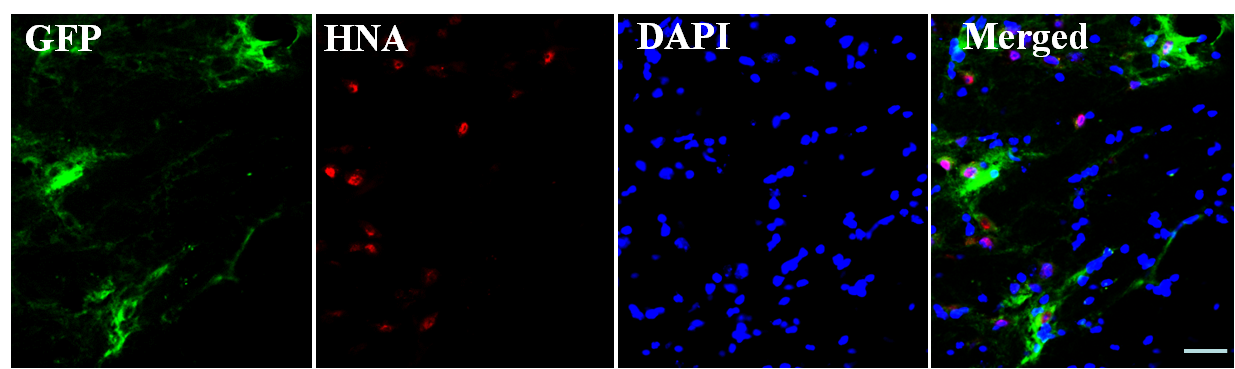

Supplement: Additional file 11: Figure S2. — Histoimmunostaining with human nuclear antigen (HNA) to trace the transplanted hADSCs in mouse MCAO model brain. [file 13287_2015_78_MOESM11_ESM.tif]
